# Angiogenesis Dynamics: A Computational Model of Intravascular Flow Within a Structural Adaptive Vascular Network

**DOI:** 10.3390/biomedicines12122845

**Published:** 2024-12-13

**Authors:** Sahar Jafari Nivlouei, Ana Guerra, Jorge Belinha, Naside Mangir, Sheila MacNeil, Christiane Salgado, Fernando Jorge Monteiro, Renato Natal Jorge

**Affiliations:** 1 INEGI—Instituto de Ciência e Inovação em Engenharia Mecânica e Engenharia Industrial, 4200-465 Porto, Portugal; s.jafari.me@gmail.com; 2 ISEP—Instituto Superior de Engenharia do Porto, Departamento de Engenharia Mecânica, Rua Dr. António Bernardino de Almeida, 431, 4249-015 Porto, Portugal; job@isep.ipp.pt; 3 Department of Urology, Hacettepe University School of Medicine, 06230 Ankara, Turkey; n.mangir@hacettepe.edu.tr; 4 Kroto Research Institute, Department of Material Science and Engineering, University of Sheffield, North Campus, Sheffield S3 7HQ, UK; s.macneil@sheffield.ac.uk; 5 i3S—Instituto de Investigação e Inovação em Saúde, Universidade do Porto, 4200-135 Porto, Portugal; csalgado@ineb.up.pt (C.S.); fjmont@ineb.up.pt (F.J.M.); 6 INEB—Instituto Nacional de Engenharia Biomédica, Universidade do Porto, 4200-135 Porto, Portugal; 7 LAETA—Laboratório Associado de Energia, Transportes e Aeronáutica, Universidade do Porto, 4200-165 Porto, Portugal; rnatal@fe.up.pt; 8 FEUP—Faculdade de Engenharia, Departamento de Engenharia Mecânica, Universidade do Porto, 4200-165 Porto, Portugal

**Keywords:** capillary network remodelling, angiogenesis evaluation, chick chorioallantoic membrane assay, flow dynamics, hybrid meshless-based method

## Abstract

Background: Understanding vascular development and the key factors involved in regulating angiogenesis—the growth of new blood vessels from pre-existing vasculature—is crucial for developing therapeutic approaches to promote wound healing. Computational techniques offer valuable insights into improving angiogenic strategies, leading to enhanced tissue regeneration and improved outcomes for chronic wound healing. While chorioallantoic membrane (CAM) models are widely used for examining fundamental mechanisms in vascular development, they lack quantification of essential parameters such as blood flow rate, intravascular pressure, and changes in vessel diameter. Methods: To address this limitation, the current study develops a novel two-dimensional mathematical model of angiogenesis, integrating discrete and continuous modelling approaches to capture intricate cellular interactions and provide detailed information about the capillary network’s structure. The proposed hybrid meshless-based model simulates sprouting angiogenesis using the in vivo CAM system. Results: The model successfully predicts the branching process with a total capillary volume fraction deviation of less than 15% compared to experimental data. Additionally, it implements blood flow through the capillary network and calculates the distribution of intravascular pressure and vessel wall shear stress. An adaptive network is introduced to consider capillary responses to hemodynamic and metabolic stimuli, reporting structural diameter changes across the generated vasculature network. The model demonstrates its robustness by verifying numerical outcomes, revealing statistically significant differences with deviations in key parameters, including diameter, wall shear stress (*p* < 0.05), circumferential wall stress, and metabolic stimuli (*p* < 0.01). Conclusion: With its strong predictive capability in simulating intravascular flow and its ability to provide both quantitative and qualitative assessments, this research enhances our understanding of angiogenesis by introducing a biologically relevant network that addresses the functional demands of the tissue.

## 1. Introduction

Understanding vascular development is crucial in scientific research. While angiogenesis is induced by mutations in malignant tumours, new vessel formation is impaired in chronic wounds, leading to further tissue damage. Chronic wounds are characterised by their inability to advance through a normal, organised healing sequence, hindering full anatomical and functional recovery [1]. However, initiating angiogenesis and establishing stable vasculature are crucial for promoting wound healing. A deficiency in new blood vessel formation is a significant challenge for clinicians, emphasising the importance of understanding the factors regulating angiogenesis to improve therapeutic approaches. Computational techniques are powerful tools that provide valuable insights into promoting angiogenesis, enhancing tissue regeneration and improving chronic wounds healing outcomes.

Sprouting angiogenesis, where new blood vessels grow from pre-existing ones, is a fundamental biological process in several physiological and pathological conditions. It plays a crucial role in organ formation, wound healing, inflammation, embryonic development, and contributes to the onset and progression of several diseases such as diabetes, rheumatoid arthritis, and cancer [2,3,4,5,6]. Vascular Endothelial Growth Factor (VEGF) is a major mediator of angiogenesis and crucial for wound healing [7]. VEGF forms concentration gradients within tissues and exists in various isoforms with different binding capacities to extracellular matrix (ECM) components [8,9]. After injury, VEGF levels initially increase and remain elevated in wound fluid [10].

VEGF activates endothelial cells (ECs) in nearby vessels, increasing vascular permeability and triggering the secretion of proteases that degrade the basement membrane of vessels [11]. This leads to the activation of ECs, which adopt the endothelial tip cell phenotype and migrate from the blood vessel into the surrounding tissue within the extracellular matrix. Adjacent stalk cells then proliferate and promote angiogenesis [12]. Furthermore, stalk cells behind the tip cell can temporally take on the tip cell phenotype, promoting sprout elongation for several minutes [13,14]. This behaviour ensures a continuous leading-edge tip cell that exerts contractile force on the matrix, remodels the matrix fibres and creates a pathway for sprout expansion.

Animal model systems like the chicken yolk-sac or chorioallantoic membrane (CAM) are widely used to study vascular development. The CAM assay is especially useful for studying angiogenesis due to its highly vascularized structure and ability to closely mimic in vivo conditions, making it ideal for investigating angiogenic processes and developing wound healing treatments. Vascular formation in the CAM is a dynamic process that occurs rapidly across multiple scales, from capillaries to main vessels, often within hours [15]. The extraembryonic organs can be easily accessed via a shell window or shell-less experiments [16]. The vascular patterns in this model, characterised by interdigitating arteries and veins, are critical for maintaining adequate blood flow and tissue oxygenation, similar to the human retina [17]. In converse, direct artery–vein connections disrupt blood flow and hinder O_2_ and CO_2_ exchange [18]. While the CAM assay allows the direct visualisation of sprouting angiogenesis, it is insufficient for quantifying parameters like blood flow rate, intravascular pressure, vessel shear stress, and diameter changes in response to stimuli. To address this, the current study develops a mathematical model of angiogenesis to better understand the system’s overall dynamics.

Terminal vascular networks dynamically adjust to meet changing physiological requirements. Blood flow induces mechanical forces, such as wall shear stress and circumferential stretch, which directly affect the endothelium. These forces contribute to the adaptive structure of the network, ensuring relatively low intravascular pressure without overburdening blood flow [19,20]. Vessels adapt structurally in response to both metabolic conditions and hemodynamic stimuli, such as intravascular pressure and shear stress, influencing network remodelling. Structural responses, such as changes in vessel diameter, have been documented in several studies [21,22].

Over the past two decades, various approaches have been employed to model angiogenesis. Early models described vascular networks as continuous density fields based on advection–diffusion–reaction equations [23,24,25], but these lacked detail on capillary network structure and key events like sprouting, branching, and lumenogenesis. To address this, agent-based models were developed to capture cellular interactions, offering a more detailed representation of angiogenesis. These discrete models have been integrated with tumour growth models to study EC–microenvironment interactions [26,27,28], though their computational cost and complexity often limit their application. Recently, hybrid models have emerged that combine discrete cell modelling with continuous modelling of diffusing solutes and the ECM [29,30,31]. These models address key aspects of sprouting angiogenesis, including EC chemotaxis, haptotaxis, anastomosis formation, tumour-induced angiogenesis, and cell phenotype determination [32,33,34,35,36].

While previous models have advanced our understanding of angiogenesis, many lack validation against a broad range of in vivo data and fail to fully incorporate hemodynamic factors. Models like those by [29,34,37,38,39] have only partially addressed flow dynamics and their interplay with vascular remodelling. Our study overcomes these limitations by integrating diverse in vivo data to provide a more comprehensive analysis of flow dynamics and their impact on vascular structural changes. We developed a two-dimensional hybrid meshless-based mathematical model to simulate sprouting angiogenesis using the in vivo CAM system. This adaptive model accounts for capillary responses to hemodynamic and metabolic stimuli, using CAM data to predict branching processes and validate numerical outcomes. Most vascular growth and remodelling occur after blood circulation begins, when the endothelium is subjected to mechanical forces, such as wall shear stress and circumferential stress. Our model simulates blood flow through the capillary network, calculates intravascular pressure distribution, and tracks flow-related events. The model demonstrates strong predictive capabilities, with both quantitative and qualitative assessments that align with the CAM assay.

## 2. Materials and Methods

This study extended our previous work [40,41,42], simulating sprouting angiogenesis in response to VEGF concentrations using a computational algorithm based on the Radial Point Interpolation Method (RPIM) combined with a VEGF diffusion formulation. EC migration and sprout branching were driven by chemotaxis. The key innovation of this study lay in modelling intravascular flow within vessels. The model not only calculated blood flow dynamics, including intravascular pressure and wall shear stress, but also introduced dynamic vascular remodelling in response to environmental cues. The resulting vascular development was validated against in vivo observations from the CAM assay.

### 2.1. Numerical Model of Angiogenesis

A meshless method was implemented to solve VEGF distribution. First, the model discretizes the physical domain using a nodal set conforms to the area of the domain. Subsequently, the background integration lattice was defined by employing quadrilateral regular cells that precisely matched the area of the domain. The numerical integration was performed using the Gauss–Legendre quadrature rule where each integration point is characterised by its Cartesian coordinates $x_{I}\in R^{2}$ and its corresponding integration weight $\hat{w_{I}}$. This collection of integration points forms the background integration mesh, allowing the discrete integration of the continuous equations that govern the underlying physical phenomenon. At this stage, the nodal connectivity can be established using the “influence-domain” concept, which refers to the set of nodes that are closer to each integration point $x_{I}$. This set of nodes construct the shape function of integration point where the weak form is then applied to formulate the system of equations. Hence, applying the RPI technique, the concentration of VEGF at any integration point is as follows:

(1) $$\phi^{h}(x_{I})=\varphi (x_{I}{)}^{T}\phi$$

where $\varphi (x_{I})$ is the shape function vector at the integration point $x_{I}$, defined as:

(2) $$\varphi (x_{I})={\left\{\varphi_{1}(x_{I})\varphi_{2}(x_{I})\ldots \varphi_{n}(x_{I})\right\}}^{T}$$

where

(3) $$\left\{\begin{array}{c} \begin{array}{c} \varphi_{1}(x_{I})\\ \varphi_{2}(x_{I}) \end{array}\\ \vdots \\ \varphi_{n}(x_{I})\\ \lambda (x_{I}) \end{array}\right\}={\left[\begin{array}{ccccc} r_{1}(x_{1}) & r_{2}(x_{1}) & \ldots & r_{n}(x_{1}) & 1\\ r_{1}(x_{2}) & r_{2}(x_{2}) & \cdots & r_{n}(x_{2}) & 1\\ \vdots & \vdots & \ddots & \vdots & \vdots \\ r_{1}(x_{n}) & r_{2}(x_{n}) & \ldots & r_{n}(x_{n}) & 1\\ 1 & 1 & \cdots & 1 & 0 \end{array}\right]}^{-1}\left\{\begin{array}{c} \begin{array}{c} r_{1}(x_{I})\\ r_{2}(x_{I}) \end{array}\\ \vdots \\ r_{n}(x_{I})\\ 1 \end{array}\right\}$$

$\lambda (x_{I})$ is a by-product of the equation system and $r_{i}(x_{j})$ the multi-quadric radial basis function as following:

(4) $$r_{i}(x_{j})={\left((x_{i}-x_{j}{)}^{2}+(y_{i}-y_{j}{)}^{2}+c^{2}\right)}^{P}$$

The parameters c and P are shape parameters controlling the interpolation property of the resulting shape function, and they were already optimised to c = 0.0001 and P = 0.9999 in previous studies. For more details, please see [43,44,45].

A discrete equation system is established to obtain the numerical solution of the algorithm implemented in the RPIM. Hence, to simulate the ECs chemotaxis regulated by the VEGF diffusion in the ECM, the Helmholtz form is used in a 2D linear steady state field, as follows:

(5) $$D_{x}\frac{\partial^{2}\phi }{\partial x^{2}}+D_{y}\frac{\partial^{2}\phi }{\partial y^{2}}+R=0$$

where ϕ corresponds to the VEGF concentration, D is the diffusion coefficient, and R is the VEGF release rate. The meshless system equations are formulated using the weighted residual approach:

(6) $$\left[K_{D}+K_{g}\right]\Phi -f_{q}=0$$

(7) $$K_{D}=\int_{A}{B_{G}}^{T}DB_{G}dA$$

(8) $$K_{g}=\int_{A}g{\left\{\begin{array}{cccc} \varphi_{1} & \varphi_{2} & ... & \varphi_{n} \end{array}\right\}}^{T}\left\{\begin{array}{cccc} \varphi_{1} & \varphi_{2} & ... & \varphi_{n} \end{array}\right\}dA$$

(9) $$f_{q}=\int_{A}Q{\left\{\begin{array}{cccc} \varphi_{1} & \varphi_{2} & ... & \varphi_{n} \end{array}\right\}}^{T}dA$$

Considering $f_{q}$ as the VEGF concentration flux, $B_{G}$ and D are defined as:

(10) $$B_{G}=\left[\begin{array}{c} \frac{\partial \varphi }{\partial x}\\ \frac{\partial \varphi }{\partial y} \end{array}\right]=\left[\begin{array}{ccc} \frac{\partial \varphi_{1}}{\partial x} & \frac{\partial \varphi_{2}}{\partial x} & \ldots \;\;\;\frac{\partial \varphi_{n}}{\partial x}\\ \frac{\partial \varphi_{1}}{\partial y} & \frac{\partial \varphi_{2}}{\partial y} & \ldots \;\;\;\frac{\partial \varphi_{n}}{\partial y} \end{array}\right]$$

(11) $$D=\left[\begin{array}{cc} D_{x} & 0\\ 0 & D_{y} \end{array}\right]$$

Finally, solving Equation (6) gives the final VEGF concentration in the domain.

To start angiogenesis, tip cells are marked and chosen based on the CAM images to reduce the number of unknown variables in the initial conditions, such as the starting sprout location. Specifically, the tip cells are identified within the sub-domain defined as the endothelial cell monolayer using Cartesian coordinates derived from the CAM images. This approach ensures a precise and biologically relevant representation of the initial sprout positions. Following the marking of the initial tip cells, the algorithm initiates an iterative loop, where at each time step, new tip cells are sequentially marked, facilitating the growth of sprouting vessels over time. Each time step in this study corresponds to the duration required for a capillary to migrate a single length of an endothelial cell. The final simulation result corresponds to the vascular pattern obtained in CAM and each iteration time corresponds to ~6.7 h. The direction of the tip cell movement follows the VEGF gradients. Accordingly, the gradient of the VEGF is obtained as n=∇Φ, which ***n*** oriented towards the VEGF source. Therefore, ${n\left[\begin{array}{cc} \cos\theta & \sin\theta \\ -\sin\theta & \cos\theta \end{array}\right]}_{mod}$ with −0.24≤θ≤0.24 attributed randomly. The new position of the tip cell is determined by adding the vector unit vector of $n_{mod}$ multiplied by the average distance between the current tip cell and the previous one to the old tip cell position. If a node already exists at the computed position of the new tip cell, it is assigned as a tip cell; otherwise, new nodes are added as newly created tip cells. In each step, the VEGF concentration is computed, and the tip cell’s position is updated accordingly.

To implement the branching process, the capillary order concept is applied according to the CAM data and simultaneously to calibrate the numerical results based on a dense capillary network. Thus, the first order of capillary represents the capillaries with higher calibre and the third order the ones with lowest. To establish the branching locations, we measured the distance between consecutive branch points for each capillary order, resulting in the following rule:

(12) $$d=0.9286e^{-0.219\times O_{cap}}$$

d represents the distance between consecutive branch points related to the capillary order $O_{\mathrm{cap}}$. Consequently, a new branch is formed within each capillary when the distance to the previous branch exceeds d, for the specific capillary order being considered. When a branch occurs, the parent capillary maintains its current order and the daughter increases its own. The average branching angle for capillaries and the function parameters are extracted from the CAM data (more details in Computational implementation). Finally, the model uses a developed phenomenological function to rule on the branching location. Then, the tip cells migrate following the highest VEGF concentration.

### 2.2. Hemodynamics and Intravascular Flow

To mimic a realistic vascular network, our study focused on the blood flow generated by sprouting, as it plays a crucial role in network formation. Assuming that the rheological parameters are known, we employed a numerical method to calculate the flow rate in each element and the pressure values at each node. Taking into account that blood flows through capillary anastomoses, we applied Poiseuille’s law to model the laminar flow:

(13) $$Q_{c}^{k}=\frac{\pi }{128}\frac{\Delta P_{b}D^{4}}{L\mu }$$

where $Q_{c}^{k}$ is the net flow rate for each capillary; c indicates the central node; $\Delta P_{b}={P_{b}}^{c}-{P_{b}}^{k}$ describes the blood pressure gradient of nodes in the vessel of diameter of D and length L; and μ is blood viscosity. Applying the volumetric flow conservation law at any interconnecting point within the network:

(14) $$\sum_{k=1}^{N}Q_{c}^{k}\beta_{k}=0$$

where k represents the selected nodes of each vessel segment and N is the total number of adjacent nodes. $\beta_{k}$ is an integer taking 0 or 1, describing whether nodes are connected or not. Using the Successive Over-Relaxation (SOR) iterative method, the intravascular pressure of each node of capillary network is numerically calculated and the flow rate in each segment is obtained.

### 2.3. Capillary Structure Adaptation

Vessels exhibit structural responses to mechanical forces generated by blood flux, specifically transmural pressure and shear stress acting on the endothelial surface [19,20]. Every segment of the network experiences a range of stimuli, which depend on the flow and metabolic conditions both within the segment itself and in other segments. Vessels adjust their diameter to maintain a specific level of wall shear stress. Hence, we investigated capillaries response to wall sheer according to the following logarithmic law, first introduced by [19]:

(15) $$S_{\tau }=\log(\tau_{w}+\tau_{\mathrm{ref}})$$

where $\tau_{w}=\frac{32\mu }{\pi D^{3}}\left|Q_{c}^{k}\right|$ and $\tau_{\mathrm{ref}}$ are small constants that are introduced to prevent singular behaviour at low wall shear stress values. Furthermore, the intravascular pressure (IP) produces an elevated stress, known as circumferential wall stress (Equation (16)) which is the main hemodynamic force acting on vessel walls and influencing arteriolar proliferation and rarefaction [22,46]. Thus, similarly to the signal from wall shear stress,

(16) $$\tau_{e}(P)=100-86\cdot \exp\left(-5000\cdot {\left[\log\left(\log\left(P\right)\right)\right]}^{5.4}\right)$$

(17) $$S_{P}=-\log\tau_{e}$$

When a particular segment of the vessel network experiences a decrease in blood flow, resulting in inadequate oxygen and metabolic material supply to the surrounding tissue, the segment needs to be stimulated to increase its diameter in order to improve perfusion. Essentially, the vessels need to adapt and expand to meet the metabolic demands of the tissue they supply. Hence, the network stability is enhanced by introducing a metabolic stimulus that triggers the growth of vessels in segments experiencing low flows:

(18) $$S_{m}=\log\left(\frac{Q_{ref}}{Q_{b}H}+1\right)$$

This is dependent on the red blood cells discharging haematocrit H and $Q_{ref}$ being the largest value of $Q_{b}$ in the network. Finally, the model predicts vessels adaption through Equation (19), by considering capillaries’ tendency to shrink ($k_{s}$) when there is no stimulating growth factor:

(19) $$\Delta D=\left[\begin{array}{c} \log(\tau_{w}+\tau_{\mathrm{ref}})-k_{p}\log\tau_{e}\\ +k_{m}\log\left(\frac{Q_{ref}}{Q_{b}H}+1\right)-k_{s} \end{array}\right]D\Delta t$$

### 2.4. In Vivo Angiogenesis

In the current work, we acquired fertilised white leghorn chicken eggs from Henry Stewart Co., Ltd. (Norfolk, UK) that were pathogens-free, and subsequently subjected them to incubation. On embryonic development day 3 (EDD3), the eggshells were carefully cracked, and the embryos were transferred into a square Petri dish. The ex ovo cultures were then kept in a humidified incubator at a temperature of 38 °C, and this environment was maintained from EDDs 3 to 14. The embryos’ survival was assessed on a daily basis and documented. For a comprehensive understanding of the ex ovo CAM assay, a detailed description can be found in a previous publication [47]. Unloaded and loaded hydrogels with an internal diameter of 8 mm were prepared (see [48] for more detail), and at EDD14, images of hydrogels and surrounding CAM area were taken using a digital microscope.

To quantify angiogenesis, three digital images from each tested condition were analysed using the NeuronJ tool (version 1.4.3), an online Image J plug-in. In CAM images, quantification was performed in 5 × 5 mm^2^ regions of interest (ROI), consistently including the biomaterial in one corner. Similarly, in the simulations, quantification was conducted in 5 × 5 mm^2^ areas, with the biomaterial consistently positioned in the lower-left corner. The tested conditions groups were unloaded, VEGF 50 ng and VEGF 100 ng and the capillary network profile also obtained from the same unloaded VEGF groups for comparison.

### 2.5. Computational Implementation

Using consistent CAM domain geometries, simulations were performed where the initial number and location of sprouting tip cells, as well as the location of the parent vessel, were identified for each simulation. According to the measured data, the average branching angle for capillaries with the same order was found to be 68° (n = 36, maximum = 127°, minimum = 45°), while for capillaries with different orders, it was 86° (n = 36, maximum = 117°, minimum = 44°). These angle measurements were incorporated into the model, allowing for some fluctuations. To simulate capillary adaptation, the diameter of the parent vessels was extracted from the CAM network and remains constant throughout the remodelling process (~0.17–0.2 mm). A no-flux boundary condition was applied to the parent vessels, while periodic boundary conditions were imposed on the opposite side of the solution domains. The pressures in arteries and veins were based on the reported experimental observations, in a range of 45 mmHg in arteries to 22 mmHg in veins [22]. The inlet and outlet pressures were selected with the aim of ensuring that the average intravascular pressure was consistent with the haematocrit, while assuming it remained constant throughout the vessels during the discharge. Parameters are listed in Table 1.

## 3. Results and Discussion

### 3.1. Angiogenesis Network

**Figure 1:**
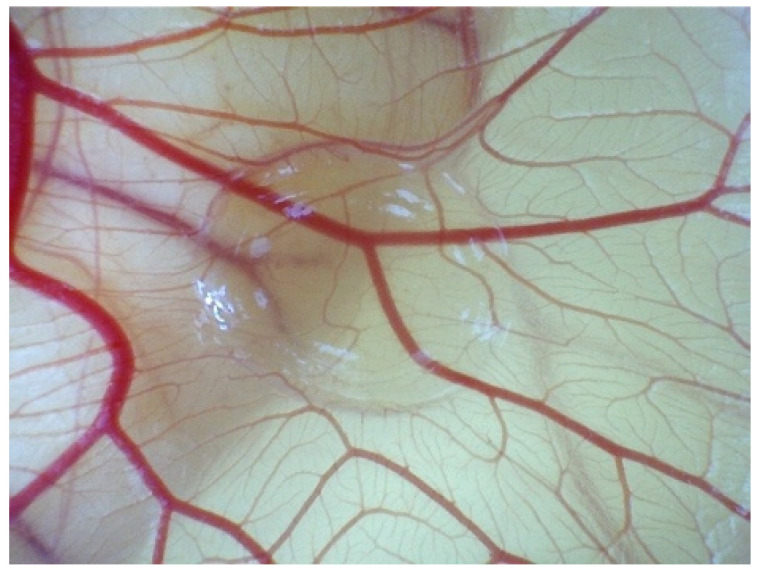
Evolution angiogenesis toward the released VEGF in the CAM assay on EDD 14.

According to in vivo, ECs migrate and self-organise towards the VEGF concentration released from hydrogels (Figure 1). To assess the angiogenic reaction resulting from chemotaxis, simulations were conducted by augmenting the VEGF concentration within the biomaterial region for the 5 × 5 mm^2^ ROI.

**Figure 2:**
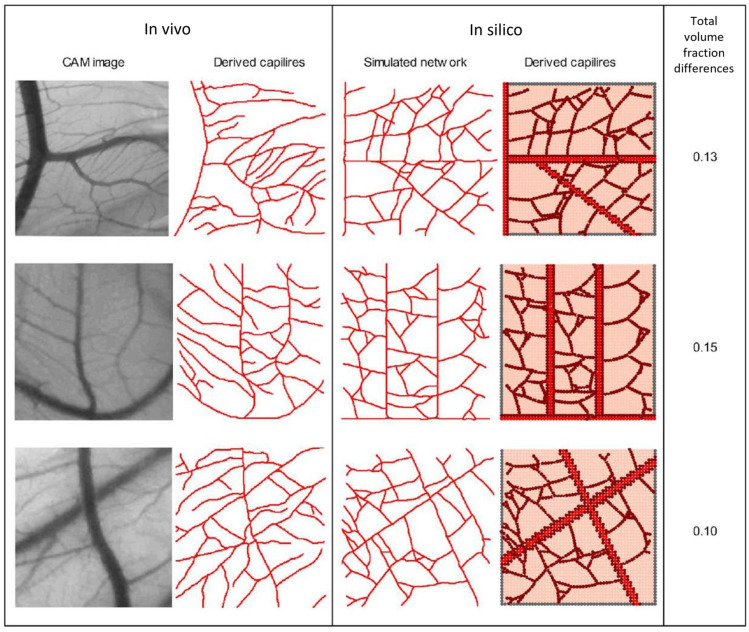
In vivo capillary network from CAM vs. In silico results, along with the corresponding total capillary volume fractions (third column).

Simulations were conducted using identical CAM domain geometries. In each simulation, the initial number and location of sprouting tip cells and the location of the parent vessel were determined. The capillary volume fraction was determined by dividing the capillary network into a 5 × 5 grid of square patches and quantifying the volume fraction in each patch to compare numerical results with in vivo data. Finally, the discrepancy between the total capillary volume fraction obtained from the CAM image and the simulation was assessed for comparison. The total capillary volume fractions obtained from CAM images and simulations showed a discrepancy of 13–15% (see Figure 2). These results indicate that the proposed model, driven solely by VEGF concentration, is capable of predicting the angiogenic response. Furthermore, in our previous research study [41], a statistical analysis was performed, revealing the model’s capability to effectively simulate the progression of sprouting angiogenesis for different VEGF concentrations.

### 3.2. Intravascular Flow

**Figure 3:**
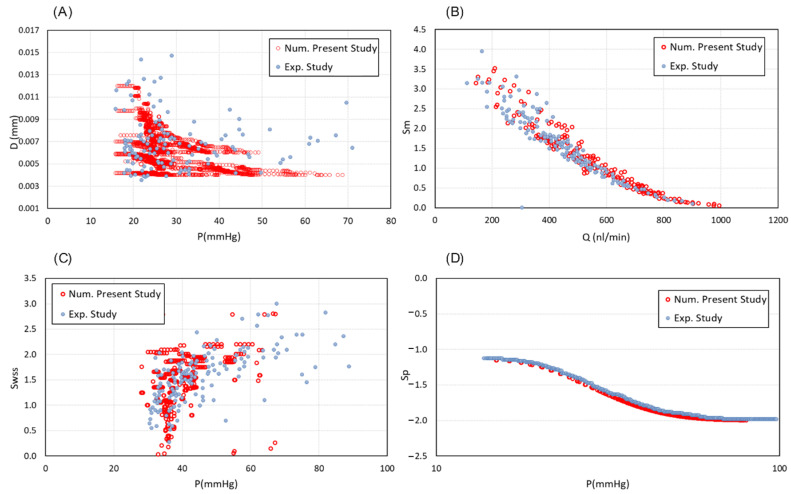
Validation of the intravascular flow modelling. (**A**) Capillary diameter changes in different values of IP in compare with experimentally determined diameters from Pries et al., 2001 [19]. (**B**) The metabolic stimuli changes as a function of flow rate inside the capillaries vs. the experimental data from Pries et al., 2001 [19]. (**C**) Distribution of hydrodynamic stimuli obtained from wall shear stress and (**D**) intravascular blood pressure, in comparison with the corresponding measured data from Pries et al., 2001 [19].

The model simulates the flow inside the capillaries to explore the impact of key parameters on the generation of a realistic vascular networks. After the formation of a closed loop (anastomosis), a pressure gradient along the vessels is created and blood flows. Anastomosis occurs when the sprouts meet and merge. To validate the obtained results, the process of vessel remodelling is employed and compared with experimental data from [19]. A broad range of pressures across various vascular network setups was analysed to capture variability within the system and provide a comprehensive assessment of flow and stress distributions under different conditions. Results demonstrate a strong agreement with the experimental observations (Figure 3). Accordingly, a reduction in intravascular pressure (IP) stimulates an increase in vessel diameter (Figure 3A). Considering the adoption stimuli distributions, the metabolic signal is higher in low-flow segments of capillaries (Figure 3B). While following Sp and Swss signals, results interpretation shows that shear stress reaches a saturation point as blood pressure increases (Figure 3C,D). Statistical analysis revealed significant differences (*p* < 0.05) were observed for diameter and wall shear stress stimuli, and highly significant differences (*p* < 0.01) were found for Sp and Sm, with a 95% confidence interval.

**Figure 4:**
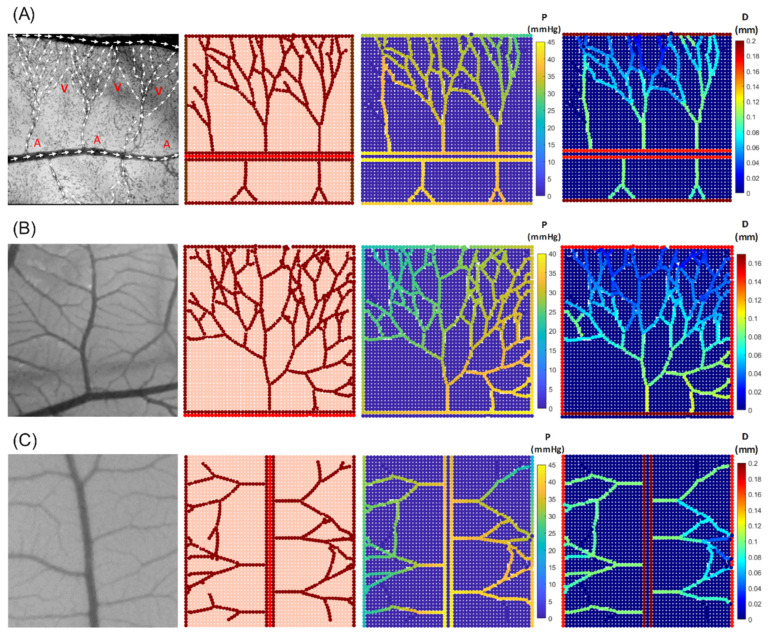
In vivo capillary network from CAM images (first column) compared with in silico results for various network configurations, illustrating intravascular pressure distribution and corresponding diameter changes. (**A**) Comparison with experimental blood flow observations reported in [51] (A—artery and V—vein). (**B**,**C**) Comparison with CAM images from the current experimental study.

A similar map is constructed based on the CAM images to represent the distributions of IP and diameter (Figure 4). Figure 4 demonstrates three distinct vascular networks, taking into account the blood flow within the capillaries. Figure 4A shows the simulated capillary network reported by [18], and Figure 4B,C are capillary profiles extracted from the current CAM images. The results indicate a slight pressure decrease along the blood flow. New vessels sprouting from the inlet region of parent capillaries maintain pressure levels similar to those of the nearby parent vessels. This contributes to the stability of the new vascular network, allowing it to accommodate high blood flow rates.

**Figure 5:**
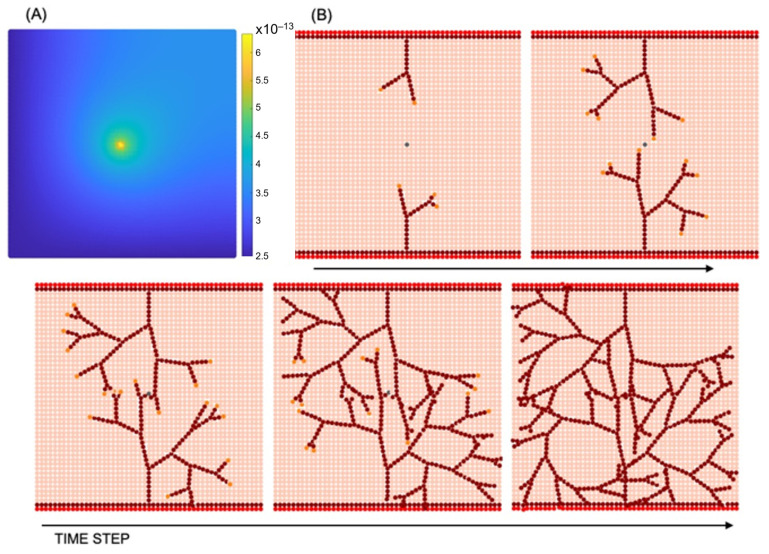
Sprouting angiogenesis: (**A**) VEGF concentration (g mm^−3^); (**B**) vascular network development resulting from tip cell migration and stalk cell proliferation in different time steps.

**Figure 6:**
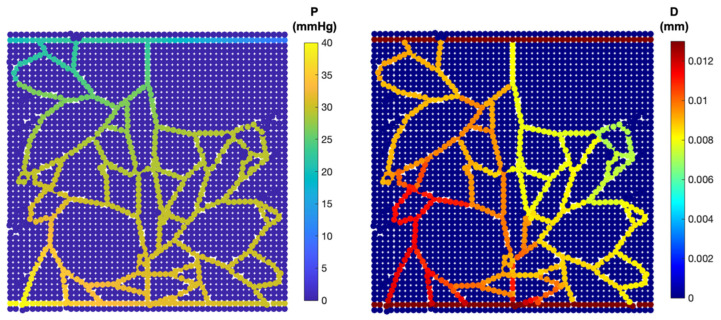
Intravascular flow: pressure distribution (**left**); diameter of each capillary segment (**right**).

For further investigation, the sprouting angiogenesis from two parent blood vessels was simulated. Notably, the network exhibits multiple anastomoses and a dense structure (Figure 5). Meanwhile, the pressure distribution and shear stress changes after the blood flow within the vessels is calculated. Results show a reduction in pressure at the distances far from the parent vessels. Similarly to the pervious findings, the neo-vessels of the inlet region maintained pressures close to those of neighbouring parent vessels (Figure 6).

**Figure 7:**
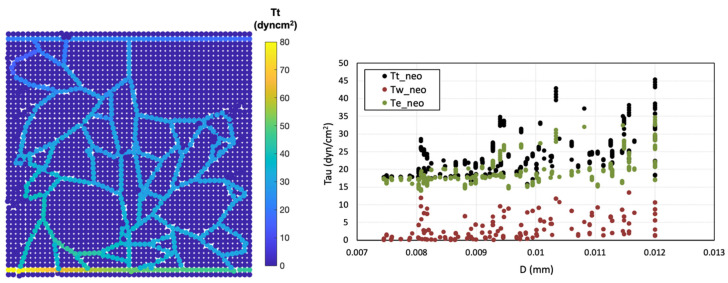
Shear stress analysis, total effective shear stress distribution ($\tau_{T}$) (**left**), shear stress changes in neo-vessels across different diameters (**right**).

Two types of shear stress—circumferential wall stress and wall shear stress—along with the total effective stress within the final network, were analysed and reported (Figure 7). The distribution of circumferential wall stress ranged from ~14–35 dyn/cm^2^, displaying a sigmoidal decrease with decreasing pressure and directly correlating with intravascular pressure. In contrast, wall shear stress was notably lower in the new capillaries, falling within the range of ~0.1–13 dyn/cm^2^. Ultimately, the total effective stress ($\tau_{T}$) is extracted since it plays a decisive role in capillary network behaviour. It provides a more accurate representation of the shear stress at the surface of ECs.

## 4. Discussion

The strong resemblance between in vivo and in silico capillary networks highlights the accuracy of the proposed VEGF-driven model in predicting angiogenic responses. The reported 13–15% discrepancy in capillary volume fractions demonstrates the model’s capability to capture essential angiogenesis processes. Although, in reality, angiogenesis is influenced by numerous other factors, the fact that the numerical model considers endothelial cell migration based solely on VEGF gradients makes these results particularly satisfactory, demonstrating that the model effectively captures and predicts angiogenic behaviour within this simplified framework. Future refinements, such as integrating additional biochemical signals and ECM heterogeneity, could improve model accuracy.

To establish blood flow and create a pressure gradient within the vessel anastomosis formation is necessary. According to the simulations, vessels anastomoses occurs when a growing sprout merges with either a functional mature vessel or another growing sprout connected to the parent vessel. Therefore, stimuli, such as the vessel wall stress and the stress induced by the local pressure, are investigated separately to analyse their role in the regulation of vessels structural adaption. We attempted to implement a vascular network based on experimental data from [18], aiming to emulate the intricate blood flow patterns observed in arteries and veins. Our primary goal was to showcase the precision of the model’s predictions when simulating blood flow dynamics. Thus, the pressure distribution is determined considering the flow direction in the created vascular network while the vessels adopt their diameters in response to environmental signals.

In terms of vascular flow, the model successfully captured critical features such as pressure gradients, shear stress distributions, and vessel remodelling. Additionally, there is an approximate decrease in diameter from proximal to distal regions driven by reduced wall shear stress ($\tau_{w}$) and increased pressure. This is attributed to the interplay of wall shear stress and pressure gradients within the vascular network. Proximally, higher stress stimulates the endothelium to maintain larger vessel diameters to accommodate higher flow rates. As the flow progresses distally, $\tau_{w}$ decreases due to cumulative branching and reduced flow velocity, while pressure increases locally. This combination drives the natural tapering of vessel diameters, optimising blood flow distribution and perfusion efficiency throughout the network.

In the context of blood flow, IP generates significant circumferential wall stress ($\tau_{e}$), which works alongside wall shear stress ($\tau_{w}$), as a major driving force through the network. Variations in pressure lead to changes in vascular morphology and network structure, with the induced shear stress playing a crucial role in arteriolar proliferation and rarefaction [22,46]. Concerning $\tau_{w}$, the flow rate, in addition to the pressure gradient of the nodes, is a crucial determining factor, clearly distinguishing it from circumferential wall stress. In fact, while the wall shear stress remains a constant for each component, $\tau_{e}$ and, consequently, $\tau_{T}$ vary due to the pressure and induce distinct values within the network. Overall, lower pressures are generally associated with decreased shear stress levels.

The integration of numerical and experimental approaches is essential, as in silico models rely on experimental data for validation and to ensure their accuracy in reflecting biological reality. In developing the current model, some simplifications were necessary to strike a balance between computational feasibility and physical relevance. The computational domain was designed to represent a realistic system while maintaining manageability in size due to the high numerical costs. Although the model does not fully capture the anisotropic and heterogeneous behaviour of the extracellular matrix (ECM) or its architectural influence on angiogenesis, it incorporates key mechanical forces, such as wall shear stress and circumferential stretch, which are critical in shaping the vascular network and maintaining efficient blood flow. Blood viscosity is assumed to be constant, and while this does not fully account for the non-Newtonian behaviour of blood in small vessels, this simplification is recognised, with its potential effect on flow dynamics noted as an area for future enhancement. These trade-offs highlight the challenges of developing computational models and lay the groundwork for future improvements.

## 5. Conclusions

This study presents a novel hybrid meshless-based mathematical model to study angiogenesis and intravascular flow, demonstrating significant advantages in its predictive abilities for both the quantitative and qualitative assessments of angiogenesis. The proposed model employs in vivo CAM data to predict the branching progression and validate numerical simulations, incorporating dynamic capillary network. The adaptive network associated with the angiogenesis enables a comprehensive consideration of capillaries response to hemodynamic and metabolic triggers, thereby enhancing the model’s validity.

Quantitatively, this model excels in predicting the interplay between intravascular pressure, shear stress, and vascular network architecture. The ability of neo-vessels to maintain pressure proximity to that of parent vessels, particularly those originating from the inlet region, highlights the model’s capacity to predict stable vascular networks capable of accommodating substantial blood flow rates. As blood circulates, shear stress is exerted upon capillaries. However, capillaries widen to relieve the stress. Normally, vasodilation occurs to decrease the vessels wall shear stress and blood pressure. However, if the blood flow rate is below a threshold, capillaries switch to vasoconstriction to increase the pressure and stress to a level that keeps the vessel stable.

The intricate relationship between pressure, shear stress, and vascular behaviour is comprehensively elucidated through the integration of two distinct shear stress types along with total effective stress. Results demonstrated that the variation in circumferential wall stress, mirroring pressure changes, emphasises its pivotal role in vascular physiology. While wall shear stress remains relatively consistent, the effective stress offers insights into capillary network dynamics, further unveiling the complex interplay between pressure, shear stress, and vessel behaviour. In general, lower pressures are correlated with reduced shear stress levels. Accordingly, the model demonstrates capillary adaptations such as vasodilation and vasoconstriction, mechanisms essential for maintaining stability in response to changes in blood flow rate and shear stress. The model’s ability to simulate such dynamic phenomena underscores its robustness in capturing key vascular behaviours.

Despite the model’s success, it does not account for the critical influence of oxygen concentration and hypoxia, factors that are essential in the angiogenesis process and play a significant role in wound healing. Hypoxia is a key driver of vascular development, influencing endothelial cell behaviour, growth factor secretion, and capillary sprouting. Incorporating oxygen gradients and hypoxic conditions into future models will enhance their biological relevance and provide deeper insights into wound healing, where oxygen availability is often a limiting factor.

Finally, our model agrees well with experimental observations of angiogenesis, accurately replicating essential aspects of angiogenesis, capillary responses, and dynamic intravascular flow patterns.

## Figures and Tables

**Table 1:** Parameters used in the model and corresponding references.

Parameters	Description	Value	References
Angiogenesis			
D	VEGF diffusion coefficient	$1.16\times 10^{-6}$ mm^2^ s^−1^	[49,50]
R	VEGF release	$500\times 10^{-6}$ g mm^−3^	[40,42]
Capillary network adaption			
$\tau_{ref}$	Reference value for shear stress	$7.73\;\times \;10^{-5}\;mmHg$	[19]
$k_{p}$	Scaling parameter for sensitivity to intravascular pressure	0.68 ± 0.04	[19]
$k_{m}$	Scaling parameter for sensitivity to metabolic signal	0.7 ± 0.06	[19]
$k_{s}$	Basal shrinking tendency coefficient	1.72 ± 0.15	[19]

## Data Availability

Data are contained within the article.
